# Underground Radiobiology: A Perspective at Gran Sasso National Laboratory

**DOI:** 10.3389/fpubh.2020.611146

**Published:** 2020-12-07

**Authors:** Giuseppe Esposito, Pasquale Anello, Marco Ampollini, Emanuela Bortolin, Cinzia De Angelis, Giulia D'Imperio, Valentina Dini, Cristina Nuccetelli, Maria Cristina Quattrini, Claudia Tomei, Aldo Ianni, Marco Balata, Giuseppe Carinci, Maurizio Chiti, Oscar Frasciello, Giovanni Cenci, Francesca Cipressa, Alex De Gregorio, Antonella Porrazzo, Maria Antonella Tabocchini, Luigi Satta, Patrizia Morciano

**Affiliations:** ^1^Istituto Superiore di Sanità (ISS), Rome, Italy; ^2^Istituto Nazionale di Fisica Nucleare (INFN) Sezione Roma 1, Rome, Italy; ^3^Laboratori Nazionali del Gran Sasso-INFN, Assergi, L'Aquila, Italy; ^4^Laboratori Nazionali di Frascati-INFN, Frascati, Italy; ^5^SAPIENZA Università di Roma, Rome, Italy; ^6^Museo storico della Fisica e Centro Studi e Ricerche “Enrico Fermi”, Rome, Italy; ^7^Independent Researcher, Rome, Italy

**Keywords:** low radiation environment, underground biology, radiobiology, *Drosophila melanogaster*, DULIA-*bio*, LNGS, RENOIR

## Abstract

Scientific community and institutions (e. g., ICRP) consider that the Linear No-Threshold (LNT) model, which extrapolates stochastic risk at low dose/low dose rate from the risk at moderate/high doses, provides a prudent basis for practical purposes of radiological protection. However, biological low dose/dose rate responses that challenge the LNT model have been highlighted and important dowels came from radiobiology studies conducted in Deep Underground Laboratories (DULs). These extreme ultra-low radiation environments are ideal locations to conduct below-background radiobiology experiments, interesting from basic and applied science. The INFN Gran Sasso National Laboratory (LNGS) (Italy) is the site where most of the underground radiobiological data has been collected so far and where the first *in vivo* underground experiment was carried out using *Drosophila melanogaster* as model organism. Presently, many DULs around the world have implemented dedicated programs, meetings and proposals. The general message coming from studies conducted in DULs using protozoan, bacteria, mammalian cells and organisms (flies, worms, fishes) is that environmental radiation may trigger biological mechanisms that can increase the capability to cope against stress. However, several issues are still open, among them: the role of the quality of the radiation spectrum in modulating the biological response, the dependence on the biological endpoint and on the model system considered, the overall effect at organism level (detrimental or beneficial). At LNGS, we recently launched the RENOIR experiment aimed at improving knowledge on the environmental radiation spectrum and to investigate the specific role of the gamma component on the biological response of *Drosophila melanogaster*.

## Introduction

Understanding the impact of natural background radiation on metabolism of living organisms is an open puzzling issue. For about 4 billion years, life on Earth has experienced differing levels of natural radiation, of cosmic and Earth origin. Environmental radiation is believed to have played a relevant role during the evolution of living organisms, contributing to the development of still poorly characterized defense mechanisms to minimize oxidative stress (1) In addition to issues of fundamental interest, these studies also have practical implications in radiation protection and medicine. The United Nations Scientific Committee on the Effects of Atomic Radiation (UNSCEAR) “*encourage*(s) *research into the mechanistic understanding of low-dose radiation action that may contribute to disease in humans*,” and fosters studies “*to assess the health risk related to the environmental radiation exposure”* (2, 3). In this framework, underground biology is a new and very exciting area of investigation. The definition is referred to as the biological studies conducted in Deep Underground Laboratories (DULs), originally built to host particle, astro-particle and nuclear physics experiments searching for rare events such as proton or neutrino-less double decays that require a very low radioactivity environment (4, 5). These unique and extreme settings are now being exploited in other disciplines, including biology with its applications in radiobiology, making it possible to investigate if and how a substantial reduction of the exposure to environmental ionizing radiation background affects living organisms.

The Gran Sasso National Laboratory (LNGS), L'Aquila, Italy, is one of the largest DUL in the world and where most biological investigations have been conducted so far. The results of the first LNGS experiment, named Pulex, showed that the permanence in the low radiation underground laboratory decreases the defense mechanisms against chemical radio-mimetic compounds in yeasts (6). More recently, the PULEX-COSMIC SILENCE experiments get more insight into the role of environmental radiation in the biological response of different *in vitro* mammalian cellular systems of rodent (7–9) and human origin (10, 11). In 2018, we obtained the first evidence of the influence of environmental radiation on the response of a complex organism, namely *Drosophila melanogaster* (12, 13).

Interestingly, similar evidence has been obtained from experiments conducted in other DULs using protozoan, bacteria, and mammalian cells (14–18).

The general message coming from all these studies is that environmental radiation may trigger biological mechanisms that increase the capability to cope against stress.

However, several issues are still open, among them: the role of the quality of the radiation spectrum in modulating the biological response, the dependence on the biological endpoint and on the model system considered, the role of genetic/epigenetic modifications (19), the overall effect at organism level (detrimental or beneficial).

The growing interest in underground radiobiology is testified by the establishment of several projects, like LBRE that is being conducted at WIPP (USA) since 2011 (15), the more recent REPAIR at SNOLAB (Canada) (20), the Deep-underground Medicine studies at CJEML (China) (21) and SELLR at Boulby Underground Laboratory (UK) (22).

This scenario has driven the organization of a series of dedicated Workshops, named DULIA-*bio* (Deep Underground Laboratory Integrated Activity in biology), with the purpose to establish a common path for European/International underground laboratories in deep life studies and its application to astrobiology, biophysics, human health and radiation protection. The first one was held in Canfranc, Spain, in October 2015,^1^ the second at the LNGS, Italy, in November 2019.^2^ The next DULIA-*bio* Workshop is expected to be organized in Boulby, UK.

In this Perspective paper, after a brief summary of the LNGS experience in conducting underground radiobiological studies, we report on our approach to get more insight in the field of extremely low dose rate radiobiology. In our opinion, a crucial point is to elucidate the role of the different components of the environmental radiation field on the response of living organisms. With this aim, we recently started the RENOIR (*Radiation ENvirOnment triggers bIological Responses in flies: physical and biological mechanisms*) experiment, using *Drosophila melanogaster* as *in vivo* model system. We will describe the challenges faced, the strategies to overcome them and the first dosimetric and biological results finalized to optimize the experimental set up.

## The Underground Radiobiology Experience at LNGS: History, Achievements and Challenges

During the construction of the LNGS, a group of physicists was involved in the measurements of the radiation field inside the experimental halls that were being built. One of them, Luigi Satta, was “*struck by the numbers,”* as he uses to say. Indeed, it was immediately clear that, thanks to the 1400 m-coverage of dolomitic rocks poor of uranium and thorium, the underground radiation field was considerably reduced with respect to the external environment. Luigi Satta's pioneering idea of conducting underground experiments with biological systems soon took shape and a multidisciplinary group of scientists launched the first biological experiment on yeasts. Since then, biological investigation has continued for many years using different *in vitro* and *in vivo* model systems and most recently the fruit fly *Drosophila melanogaster*.

Although *in vitro* studies are continuing to provide important mechanistic answers, *in vivo* investigation remains a top priority for the underground radiobiology community seeking a “robust” model organism suitable for studies in these extreme environments.

In our experience, *Drosophila melanogaster* has proven to be particularly responsive to changes in environmental radiation promptly modifying its physiological responses. However, in addition to flies, other organisms are currently being investigated, namely fishes at the SNOLAB (23) and nematodes at WIPP (24). The comparison between the responses of the organisms placed at different levels of the phylogenetic tree will provide interesting insights to understand the role of natural radiations in the adaptation and evolution of living organisms.

Since the pilot yeast study, the LNGS experience demonstrated that a fruitful approach is to conduct parallel tests in underground Low Radiation Environment (LRE) and in above ground Reference Radiation Environment (RRE), keeping working conditions and environmental parameters as much as possible the same, except for the ionizing radiation background. Since the visible solar spectrum at LRE can influence the circadian rhythms of flies, we decided to eliminate this source of variability using two identical *Drosophila* incubators (one at LRE and the other at RRE) in which flies live under the same conditions of light, temperature and humidity.

A close monitoring of the experimental parameters is mandatory for the interpretation of the results. As for the biological tests, reagents from the same batch should always be used. Furthermore, the presence of specialized facilities, e.g., Mechanical Workshop, Electronics, Chemistry & Chemical Plants Services, can be extremely useful for any experimental need.

LNGS, being located in a tunnel under a mountain, has some favorable features. There is no significant difference in terms of atmospheric pressure; moreover, an easy horizontal access to the LRE and the proximity of the RRE and the LRE labs allows the same operator to conduct measurements in both locations during the same day, minimizing other possible sources of variability.

Over many years of researches, the multidisciplinary Cosmic Silence Collaboration has developed and improved strategies and implemented facilities to further optimize the working conditions at LNGS. The new Cosmic Silence facility dedicated to *in vivo* studies was recently built and equipped, next the Pulex one, dedicated to *in vitro* studies. A key goal of the research group is to continuously improve the characterization of the radiation field in the environments where the experiments are conducted. In particular, during the biological tests, radon is continuously monitored as well as the gamma dose-rate. Furthermore, especially designed devices are under implementation to modulate the gamma component, in order to evaluate this specific contribution on the observed biological responses (see below “The RENOIR Experiment”).

## The Renoir Experiment: A First Step In the Evaluation of the Role of Radiation Quality

It is interesting to note that *Drosophila melanogaster*, well-known to be a radioresistant organism, responds so promptly to changes in the environmental radiation. The doses/fluences of concern are so low that we should speculate about the triggering of bystander mechanisms, typical of the so-called ≪non-targeted effects≫, that involve cell-cell communication phenomena for amplifying such small signal(s) (25).

In the attempt to understand whether the biological response is related to an overall increase of the dose-rate exposure or to the contribution of specific component(s) of the radiation field, the three-year RENOIR experiment recently started at LNGS. We adopt a step by step approach; the first phase will consists in modulating the gamma component using shielding or naturally occurring gamma-emitters.

RENOIR has two main aims: 1. to improve the knowledge of the radiation field at RRE and at LRE; 2. to obtain information about the involvement of the gamma component of the environmental radiation field on the fertility and gene expression of fruit flies.

### Characterization of the Radiation Field

Different components contribute to the overall environmental dose/dose rate in the different experimental sites. As evaluated on the basis of the UNSCEAR Report (26), at RRE the cosmic rays (mostly muons) contribution to the dose rate is of about 41 nSv/h, that of cosmic-ray neutrons of about 21 nSv/h; the terrestrial gamma rays contribution, measured by LNGS colleagues, is of about 22 nSv/h (Di Carlo personal communication). At LRE, the dose rates due to cosmic rays and neutrons are strongly reduced with respect to those at RRE (27), while the gamma dose rate is comparable (about 20 nSv/h, as obtained by us in Reuter Stokes, Automess and TLD measurements).

Another contribution to the environmental dose/dose rate comes from the radon (^222^Rn) decay products. In the underground Pulex-Cosmic Silence facilities, an efficient ventilation system is running to maintain radon concentration comparable to that at RRE. Hourly and daily variations of indoor radon activity concentration in air are routinely monitored by the AlphaGUARD active device. We are currently able to have average values of ~15 Bq/m^3^ and ~20.43 Bq/m^3^, at LRE and RRE, respectively.

One of the first goals of the RENOIR Project is to further characterize the radiation field by semi-quantitative *in-situ* gamma spectroscopic measurements using a high-efficiency commercial portable spectrometer (80% HpGe). With this technique, already successfully applied in indoor environments since the 80' (28, 29), we will collect and elaborate the gamma spectra to gain information about the gamma fluxes in the experimental sites. Moreover, the gamma dose rates will be measured at RRE and LRE laboratories with organic scintillator dose rate meters, Reuter Stokes high pressure ionization chamber and high sensitivity thermo-luminescent detectors (TLD).

To detect thermal neutrons, we will subtract the gamma contribution measured by TLD-700H from the TLD-600H gross reading, these latter also exhibiting a large thermal neutron absorption cross section. To perform a dosimetric and spectroscopic investigation of the neutron field in a wider energy range, we will use a BF3 proportional tube in particular a Boron trifluoride detector (Centronic) and the DIAMON spectrometer, a low energy resolution neutron spectrometer developed by the Nuclear Measurements group (Energy department, PoliMi, Italy) in collaboration with RAYLAB (30).

Finally, in order to characterize radiation field components that are not directly detectable by our instrumentations (e.g., muons) and to arrange a suitable dose model inside both LRE and RRE, we will conduct Monte Carlo simulations. Simulations will also help in the design and realization of the devices we are implementing for decreasing or increasing the gamma dose rate. Indeed, the modulation of the gamma component of radiation field is linked to the second major goal of RENOIR.

### Implementation and Optimization of Devices for Modulating the Gamma Component of the Radiation Spectrum

In order to reduce of several hundred times the gamma component at RRE, we are adapting to our purposes a Gamma spectrometry shield. The 10 cm thick lead hollow cylinder will be equipped with a properly designed ventilation system to prevent the accumulation of radon and with a temperature and light control system for proper *Drosophila* maintenance.

To increase the gamma component at LRE, we will use especially designed Marinelli beakers filled with natural gamma-emitter building material (tuff and pozzolana), and sealed to avoid any radon exposure. This approach has been proposed for the first time by the WIPP group who used gamma from ^40^K to increase the dose rate underground in bacteria experiments (16).

**Figure 1:**
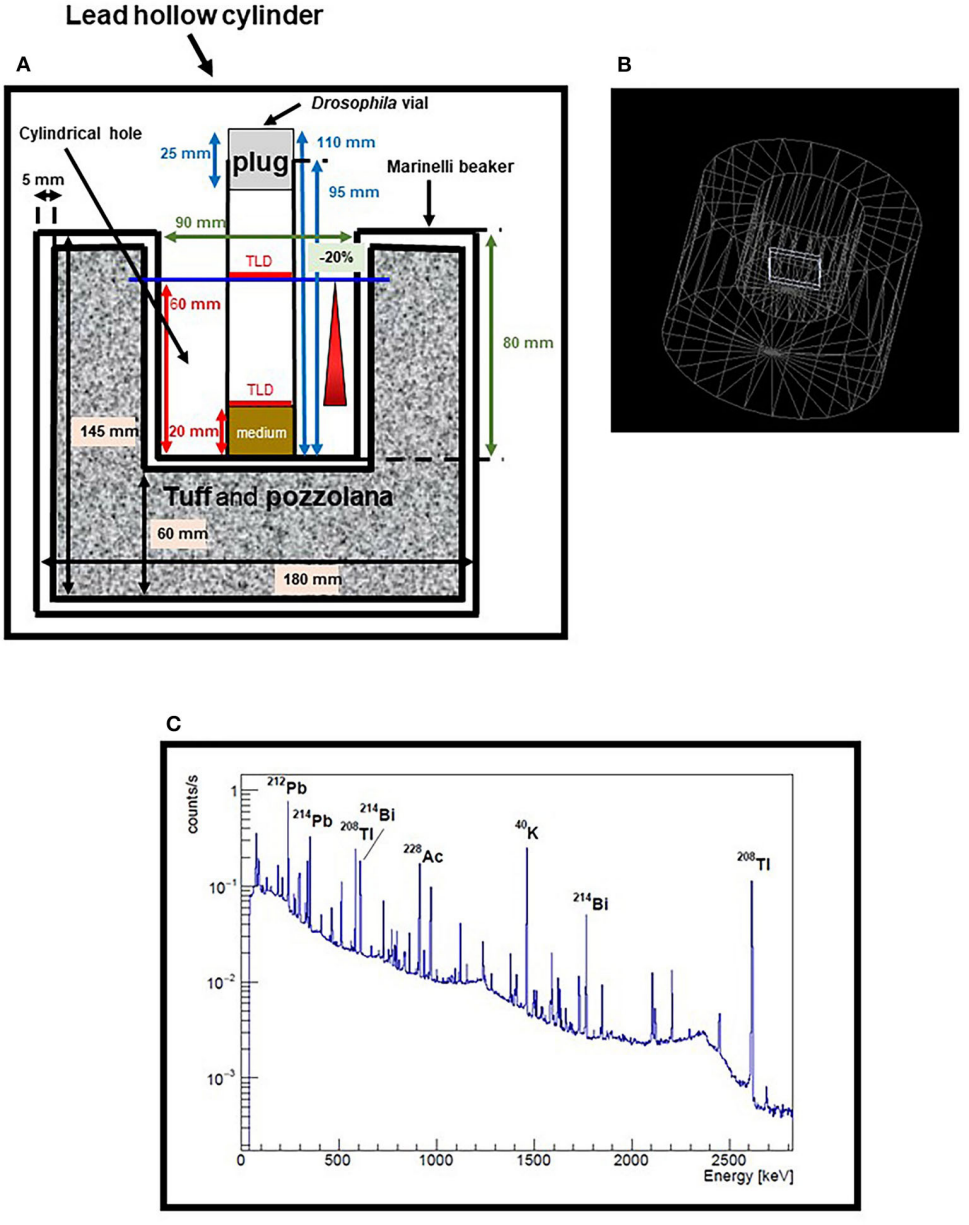
Measurements and simulations for the optimization of the Marinelli beaker. **(A)** Scheme of Marinelli beaker currently in use, placed inside the 10 cm thick lead hollow cylinder. Only the *Drosophila* vial in the center of the cylindrical hole is shown, with two sets of TLDs positioned at two different heights. Dimensions of Marinelli are in black, dimensions of *Drosophila* vial are in blue, dimensions of the cylindrical hole are in green and the heights of TLDs are indicated in red. **(B)** Geometry of the Marinelli beaker implemented in the simulation. The TLDs were simulated with dimensions 40 × 40 × 4 mm^3^, that is with the same thickness but much wider surface area than the real detectors, to get a reasonable compromise between statistics and computation time. **(C)** Spectrum of a tuff/pozzolana sample, measured with an HPGe detector. Some of the major peaks are shown as examples of the heterogeneity of the emitters in the sample.

The increase of the dose rate with the gamma rays from the decay of the different emitters present in our tuff/pozzolana mix (see Figure 1C) is a first step in the attempt to simulate the low LET component of the background radiation spectrum. Starting from the measured value of about 20 nGy/h for gamma rays at LRE (i.e., the only low LET radiation type in this environment), we will gradually increase this contribution up to the total dose rate value of the low LET components at RRE (i.e., gamma rays plus muons) and even beyond. We will use TLD to monitor the dose rate inside and outside the shield and the Marinelli beaker.

Presently, we have launched a series of gamma radiation measurements using TLD-700H inside the cylindrical hole of a large size standard Marinelli beaker placed into the 10 cm thick lead cylinder (Figure 1A). A first set of measurements was performed during a period of 41 days with the aim to verify the vertical and horizontal variations in the dose distribution experienced by flies. Several vials containing five TLDs each, were placed at the perimeter of the Marinelli internal hole, at the same height. An additional vial was placed in the center of the hole in which two sets of TLDs were positioned at two different heights, i.e., 20 mm e 60 mm respectively (representing the base of the nutrient medium and the max height reached by the flies). Preliminary results gave a dose rate of ~122 nGy/h, with a variation coefficient within 2.5% for the dosimeters placed in the same plane and a difference of about 20% between the dosimeters located at the two different heights. In order to reduce this difference below 10%, a value acceptable to our purposes, we decided to design new Marinelli beakers.

Simulations by means of the Geant 4 code (31) were performed where the geometry of the Marinelli beaker was implemented and its dimensions parameterized, keeping the internal hole fixed (Figure 1B). The volume of the Marinelli was assigned a material with the chemical composition and density of a standard rock. Then, gamma rays were generated and uniformly distributed within this volume, with isotropic direction distribution and energy randomly extracted from a spectrum of a tuff/pozzolana sample, as measured by an HPGe detector (Figure 1C).

As expected, the variation between the dosimeters located at the two aforementioned heights was mainly due to the irradiation coming from the bottom part of the Marinelli. Therefore, we simulated only the upper part of the Marinelli, a cylindrical shell with an inner and outer diameter of 90 mm and 180 mm, respectively, and performed a scan of the simulated dose rate by varying the position of the TLD. We found that in the central region between 20 and 60 mm the dose rate is constant within 5%, a condition that is suitable for our purposes.

Simulations also indicate that increasing the outer diameter to 280 mm, the dose rate at the position of 20 mm height could be increased up to 40%.

### Feasibility Test of *Drosophila* Irradiation Using Marinelli Beaker

**Figure 2:**
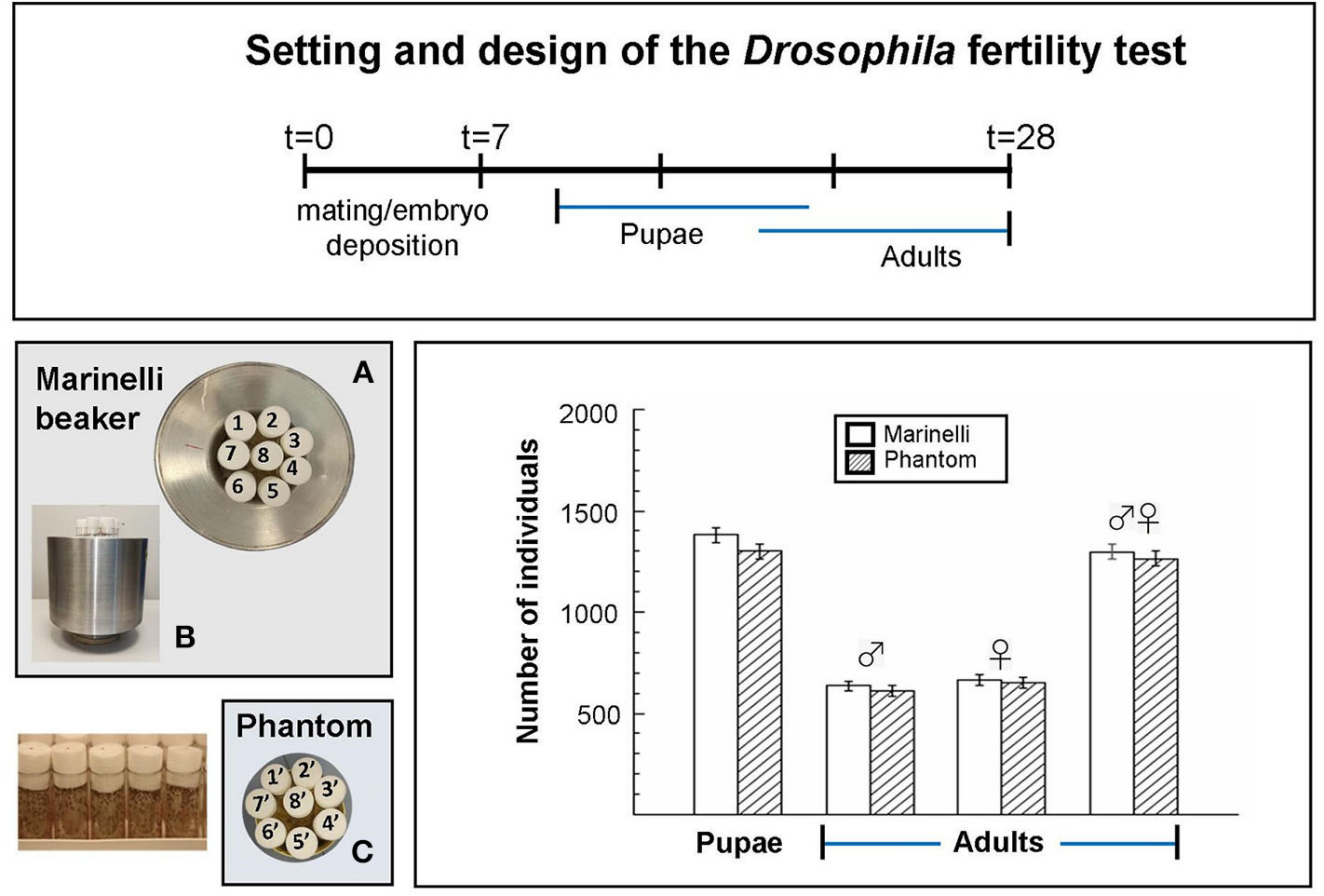
Benchmark *Drosophila* fertility test, carried out in the reference external LNGS laboratory, aimed at checking the irradiation configuration and getting information for optimizing the design of the new device. Setting and design of the *Drosophila* fertility test: After mating and embryo deposition, pupae and adults were counted for the periods indicated by the blue lines. **(A)** Top view and **(B)** lateral view of eight tubes containing flies inside the Marinelli; **(C)** top view of eight tubes containing flies inside the phantom. Columns indicate the total number of pupae and adults (males and females) from five replicates (tubes) obtained in three independent experiments. Error bars represent the square root of the total counts. Statistical differences between the results obtained inside and outside the Marinelli beaker were analyzed using the Student's t test (*p* < 0.05). No significant differences were observed.

Taking advantage of our previous results (12), to test the Marinelli as exposure device we conducted at RRE a benchmark test on fertility, being this end point robust and less time consuming than life span. Wild-type *Drosophila* was put inside the Marinelli beaker and also inside a phantom, having the same geometry as the Marinelli, in the absence of naturally occurring gamma-emitters. Both were placed on the bench in the same fly-room two meters apart each other in order to significantly reduce the gamma irradiation from Marinelli to the phantom. Temperature, relative humidity and day/night light cycle were the same. Briefly, five young wild-type males and five virgin wild-type females were crossed for 1 week, parents were removed and progeny was left to develop to adults. The number of pupae was counted along with the number of hatched adults until 21 days. No significant differences were observed in the number of pupae or hatched adults per cross in the two exposure scenarios (Figure 2), suggesting that *Drosophila* fertility is not affected by ~5 times increasing of the gamma component at RRE (~122 nGy/h vs. ~22 nGy/h).

The experiment was useful to check the irradiation configuration and confirmed the feasibility of the test in the conditions we are setting.

## Conclusions

Underground biology is becoming a field in constant and considerable expansion. The last few years showed a growing interest from several worldwide DULs for biological studies and increasing efforts to implement in loco underground radiobiology. Although a limited number of research groups are involved in this field of investigation, due to the limited number of DULs worldwide and the difficulties of working in such extreme places, the information that comes from controlled studies in underground environments can be extremely interesting from basic and applied science.

Experiments conducted in DULs have shown changes compared to above ground laboratories in the responses of bacteria, protozoa and mammalian cells, as well as in more complex organisms, i.e., flies, fishes and worms. Experimental evidence indicates that despite natural radiation background is presently extremely small nevertheless it may be significant enough for living organisms to sense it and respond to it, keeping memory of this continuous exposure.

Moreover, observation of a lower stress response below the average environmental background, associated to the non-linear responses observed at low dose/dose rate, further challenges the radioprotection assumption that stochastic risk is directly proportional to dose (the LNT model) (32, 33).

The search for valid multicellular model systems and robust endpoints, the definition of efficient strategies and the need of deep radiation environment characterization for radiation quality studies are some of the objectives pursued by the underground biology groups (DULIA-bio community).

In this framework, RENOIR's results are expected to help take a step forward this direction, especially if properly integrated with data coming from above ground laboratories at increasing dose rates.

To conclude, investigation in DULs can help to understand the molecular mechanisms underlying biological effects observed at very low radiation dose/dose-rate (e.g., adaptive response and bystander effect), their interrelationship, their dependence on radiation type, total dose and dose rate and, even more importantly, their possible role in human health risks.

## Data Availability Statement

The original contributions presented in the study are included in the article/supplementary materials, further inquiries can be directed to the corresponding author/s.

## Author Contributions

GE, MT, and PM designed and wrote the article. GE, CT, and PM designed the figures. PM performed the *Drosophila* experiments. CT and GD'I performed simulations. MA, EB, CD, CN, and MQ designed and performed dosimetric analysis. GE performed biological data statistical analysis. All the authors contributed to manuscript editing, revision and approved the submitted version.

## Conflict of Interest

The authors declare that the research was conducted in the absence of any commercial or financial relationships that could be construed as a potential conflict of interest.

## Footnotes

^1^https://indico.cern.ch/event/436589/overview

^2^https://agenda.infn.it/event/19116/timetable/

## References

[1] Lampe N Breton V Sarramia D Sime-Ngando T Biron DG. Understanding low radiation background biology through controlled evolution experiments. Evol Appl. (2017) 10:658–66. 10.1111/eva.12491 28717386 PMC5511359

[2] UNSCEAR Biological Mechanisms of Radiation Actions at Low Doses. A White Paper to Guide the Scientific Committee's Future Programme of Work. New York, NY: United Nations (2012).

[3] UNSCEAR. Sources, Effects and Risks of Ionizing Radiation. UNSCEAR 2017. Report to the General Assembly. Scientific Annexes A and B. New York, NY: United Nations (2018).

[4] Smith NJT The development of deep underground science facilities. Nuclear Phys B. (2012) 229–232, 333–341. 10.1016/j.nuclphysbps.2012.09.052

[5] Best A Caciolli A Fülöp Z Gyürky G Laubenstein M Napolitani E Underground nuclear astrophysics: why and how (Review). Eur. Phys. J. A. (2016) 52:72 10.1140/epja/i2016-16072-7

[6] Satta L Augusti-Tocco G Ceccarelli R Esposito A Fiore. Low environmental radiation background impairs biological defence of the yeast *Saccharomyces cerevisiae* to chemical radiomimetic agents. Mutat Res. (1995) 347:129–33. 10.1016/0165-7992(95)00031-3 7565903

[7] Antonelli F Belli M Sapora O Simone G Sorrentino E Tabocchini MA Radiation biophysics at the Gran Sasso laboratory: influence of a low background radiation environment on the adpative response of living cells. Nuclear Phys B Proc Suppl. (2000) 87:508–9. 10.1016/S0920-5632(00)00735-0

[8] Satta L Antonelli F Belli M Sapora O Simone G Sorrentino E. Influence of a low background radiation environment on biochemical and biological responses in V79 cells. Radiat Environ Biophys. (2002) 41:217–24. 10.1007/s00411-002-0159-2 12373331

[9] Fratini E Carbone C Capece D Esposito G Simone G Tabocchini MA. Low-radiation environment affects the development of protection mechanisms in V79 cells. Radiat Environ Biophys. (2015) 54:183–94. 10.1007/s00411-015-0587-4 25636513

[10] Carbone MC Pinto M Antonelli F Amicarelli F Balata M Belli M. The cosmic silence experiment: on the putative adaptive role of environmental ionizing radiation. Radiat Environ Biophys. (2009) 48:189–96. 10.1007/s00411-008-0208-6 19169701

[11] Carbone MC Pinto M Antonelli F Balata M Belli M Devirgiliis LC Effects of deprivation of background environmental radiation on cultured human cells. Nuovo Cimento Della Soc Italiana Fisica B Basic Topics Phys. (2010) 125:469–77. 10.1393/ncb/i2010-10889-y

[12] Morciano P Iorio R Iovino D Cipressa F Esposito G Porrazzo A. Effects of reduced natural background radiation on Drosophila melanogaster growth and development as revealed by the FLYINGLOW program. J Cell Physiol. (2018) 233:23–9. 10.1002/jcp.25889 28262946

[13] Morciano P Cipressa F Porrazzo A Esposito G Tabocchini MA Cenci G Fruit flies provide new insights in low-radiation background biology at the INFN underground Gran Sasso national laboratory (LNGS). Radiat Res. (2018) 190:217–25. 10.1667/RR15083.1 29863430

[14] Planel G Soleilhavoup JP Tixador R Croute F Richoilley G Demonstration of a Stimulating Effect of Natural Ionizing Radiation and of Very Low Radiation Doses on Cell Multiplication. Vienna: International Atomic Energy Agency (IAEA) (1976).

[15] Smith GB Grof Y Navarrette A Guilmette RA. Exploring biological effects of low level radiation from the other side of background. Health Phys. (2011) 100:263–5. 10.1097/HP.0b013e318208cd44 21595063

[16] Castillo H Schoderbek D Dulal S Escobar G Wood J Nelson R. Stress induction in the bacteria *Shewanella oneidensis* and *Deinococcus radiodurans* in response to below-background ionizing radiation. Int J Radiat Biol. (2015) 91:749–56. 10.3109/09553002.2015.1062571 26073528

[17] Castillo H Li X Schilkey F Smith GB. Transcriptome analysis reveals a stress response of Shewanella oneidensis deprived of background levels of ionizing radiation. PLoS ONE. (2018) 13:e0196472. 10.1371/journal.pone.0196472 29768440 PMC5955497

[18] Liu J Ma T Liu Y Zou J Gao M Zhang R. History, advancements, and perspective of biological research in deep-underground laboratories: a brief review. Environ Int. (2018) 120:207–14. 10.1016/j.envint.2018.07.031 30098554

[19] Belli M Tabocchini MA. Ionizing radiation-induced epigenetic modifications and their relevance to radiation protection. Int J Mol Sci. (2020) 21:5993. 10.3390/ijms21175993 32825382 PMC7503247

[20] Thome C Tharmalingam S Pirkkanen J Zarnke A Laframboise T Boreham DR. The REPAIR project: examining the biological impacts of sub-background radiation exposure within SNOLAB, a deep underground laboratory. Radiat Res. (2017) 188:470–4. 10.1667/RR14654.1 28723273

[21] Xie HP Liu JF Gao MZ Wan XH Liu SX Zou J. The research advancement and conception of the deep-underground medicine. Sichuan Da Xue Xue Bao Yi Xue Ban. (2018) 49:163–8. 29737053

[22] Wadsworth J Cockell CS Murphy A St J Nilima A Paling S There's plenty of room at the bottom: low radiation as a biological extreme. Front Astron Space Sci. (2020) 7:50 10.3389/fspas.2020.00050

[23] Pirkkanen J Zarnke AM Laframboise T Lees SJ Tai TC Boreham D R A research environment 2 km deep-underground impacts embryonic development in lake whitefish (*Coregonus clupeaformis*). Front Earth Sci. (2020) 8:327 10.3389/feart.2020.00327

[24] Van Voorhies WA Castillo HA Thawng CN Smith GB. The phenotypic and transcriptomic response of the caenorhabditis elegans nematode to background and below-background radiation levels. Front Public Health. (2020) 8:581796. 10.3389/fpubh.2020.581796 33178665 PMC7596186

[25] Campa A Balduzzi M Dini V Esposito G Tabocchini MA. The complex interactions between radiation induced non-targeted effects and cancer. Cancer Lett. (2015) 356:126–36. 10.1016/j.canlet.2013.09.030 24139968

[26] UNSCEAR Sources and Effects of Ionizing Radiation. UNSCEAR 2008 Report. Vol. I New York, NY: United Nations (2008).

[27] Best A Görres J Junker M Krat KL Laubenstein M Long A Low energy neutron background in deep underground laboratories. Nuclear Instrum Methods Phys Res A. (2016) 812:1–6. 10.1016/j.nima.2015.12.034

[28] Miller KM A Spectral Stripping Method for a Ge Spectrometer Used for Indoor Gamma Exposure Rate Measurements. Report EML-419. New York, NY: Environmental Measurement Laboratory, USDOE (1984). 10.2172/6984023

[29] Miller KM Beck HL Indoor gamma and cosmic ray exposure rate measurements using a Ge spectrometer and pressurised ionisation chamber. Radiat Prot Dosim. (1984) 7:185–9. 10.1093/rpd/7.1-4.185

[30] Pola A Rastelli D Treccani M Pasquato S Bortot D DIAMON: a portable, real-time and direction-aware neutron spectrometer for field characterization and dosimetry. Nuclear Instrum Methods Phys Res. (2020) 969:164078 10.1016/j.nima.2020.164078

[31] Agostinelli S Allison J Amako K Apostolakis J Araujo H Arce P Geant4—a simulation toolkit. Nucl Instr Meth A. (2003) 506:250–303. 10.1016/S0168-9002(03)01368-8

[32] Morgan FW Bair WJ. Issues in low dose radiation biology: the controversy continues. A Perspect Radiat Res. (2013) 179:501–10. 10.1667/RR3306.1 23560636

[33] Morgan WF Sowa MB. Non-targeted effects induced by ionizing radiation: mechanisms and potential impact on radiation induced health effects. Cancer Lett. (2015) 356:17–21. 10.1016/j.canlet.2013.09.009 24041870

